# Effect of Blood Gel Derivatives on Wound Healing in Mouse Injured Tissue Models

**DOI:** 10.3390/gels9100785

**Published:** 2023-09-28

**Authors:** Tuyet Thi Vi Le, Hoang Minh Lam, My Thi Ngoc Nguyen, Nghia Thi Hieu Phan, Trang Nguyen Khanh Huynh, Hien Nguyen Trong Le, Chau Thi Hai Pham, Van Kim Hoang Tang, Trang Thi Thuy Hoang, Tuyet Thi Diem Hoang, Ha Le Bao Tran

**Affiliations:** 1 Department of Physiology and Animal Biotechnology, Faculty of Biology and Biotechnology, University of Science, VNU-HCM, Ho Chi Minh City 700000, Vietnam; ltvtuyet@hcmus.edu.vn (T.T.V.L.); ntnmy@hcmus.edu.vn (M.T.N.N.); phtnghia@hcmus.edu.vn (N.T.H.P.); 2 Laboratory of Tissue Engineering and Biomedical Materials, University of Science, VNU-HCM, Ho Chi Minh City 700000, Vietnam; lmhoang@hcmus.edu.vn; 3 Vietnam National University, Ho Chi Minh City 700000, Vietnam; 4 Hung Vuong Hospital, Ho Chi Minh City 700000, Vietnam; tranghnk08@gmail.com (T.N.K.H.); bstronghien@gmail.com (H.N.T.L.); haichaubvhv@gmail.com (C.T.H.P.); tangkimhoangvan@gmail.com (V.K.H.T.); hoangthithuytrang201190@gmail.com (T.T.T.H.); tuyethoang05@gmail.com (T.T.D.H.)

**Keywords:** growth factor, platelet lysate, platelet-rich fibrin lysate, pinopode, wound healing

## Abstract

Several previous studies in the field of assisted reproduction have focused on the use of blood gel derivatives, such as platelet-rich fibrin (PRF), as a treatment for endometrial rehabilitation. However, the ability to release growth factors and the gel form of this product led to the evolution of platelet lysates. In this study, blood gel derivatives, including PRF lysate, which was in liquid form, and PRF gel, were collected and evaluated for growth factors. It was shown to be effective in endometrial wound healing and regeneration in mouse injured uterine tissue models through structure and function (pinopode expression, embryo implantation) evaluation. The results demonstrated that the concentrations of growth factors, including PDGF-AB and VEGF-A, were higher in the PRF lysate compared to the PRF gel (*p* < 0.05). PRF lysate could release these growth factors for 8 days. Furthermore, both PRF gel and PRF lysate restored the morphology of injured endometrial tissues in terms of luminal and glandular epithelia, as well as uterine gland secretory activity. However, the presence of pinopodes and embryonic implantation were only observed in the PRF lysate group. It can be concluded that PRF lysate promotes wound healing in mouse injured tissue models in vitro, which can act as healing products in tissue repair.

## 1. Introduction

Embryo implantation is a crucial step in animal and human reproduction because it affects how successfully a pregnancy develops. A complicated interaction between the embryo and the uterus is involved in this complex process. According to extensive study, a number of growth factors and cytokines that activate complex cellular signaling pathways are necessary for the implantation process. It is probable that these mechanisms are subject to spatial and temporal regulation, occurring within both the embryo and the uterus [1].

The uterine endometrium plays a crucial function in assisting the process of embryo implantation and providing necessary support for the development and maintenance of pregnancy. The presence of endometrial dysfunction has been associated with the potential consequences of infertility [2]. Intrauterine adhesion, sometimes referred to as Asherman’s syndrome, is a widespread etiological factor contributing to infertility in the uterus. Recent research has produced encouraging findings on the prevention of recurrent intrauterine adhesion and the improvement of pregnancy outcomes through endometrial regeneration [3]. One strategy includes the utilization of mesenchymal stem cells [4,5]. Nevertheless, the proliferation of stem cells necessitates a substantial allocation of resources and an enormous amount of time.

Platelet-rich fibrin (PRF) is a blood gel derivative that has the capability to release growth factors to promote migration, attachment, proliferation, differentiation, and the synthesis of extracellular matrix [6,7]. It is an improvement over conventionally generated platelet-rich plasma (PRP) [8]. Platelet-rich fibrin (PRF) is characterized by a platelet content that is four to five times higher than that found in normal blood [9]. PRF is obtained through a short centrifugation process of whole blood without the use of anticoagulants or bovine platelet activators. Notably, PRF contains a rich reservoir of growth factors, including transforming growth factor beta 1 (TGF-β1), platelet-derived growth factor (PDGF), epidermal growth factor (EGF), fibroblast growth factor (FGF), and vascular endothelial growth factor (VEGF). These growth factors have been reported to play a crucial role in promoting angiogenesis and endometrial cell proliferation within the damaged endometrium [10]. By accurately simulating the wound healing process over an extended period of time, PRF enhances tissue regeneration by elevating the local concentration of growth factors in a particular tissue region. PRF was widely accepted for its benefits in oral and maxillofacial surgery [11]. It was also successfully treated for intrabony periodontal problems [12,13]. Furthermore, recent controlled clinical trial data have demonstrated that PRF treatment can help with musculoskeletal injuries [14]. 

Fibrin, a vital component of PRF, plays a crucial role in providing a matrix structure that facilitates wound healing, cell attachment, and angiogenesis. Consequently, it serves as an optimal scaffold material for tissue regeneration. In relation to the previous points discussed, the following aspects are addressed: (1) Physical barrier: PRF is characterized by its light-yellow gel consistency with elasticity and toughness, ensuring strong attachment to uterine wounds. Its excellent adaptability to uterine cavities of varying shapes allows for lubrication and physical support without excessive compression of the endometrium, thereby minimizing the risk of adhesions. Additionally, no instances of PRF detachment were observed in the present study. This may be attributed to the solid morphology of PRF being obstructed by the retracted cervical muscular layer following hysteroscopy removal, impeding its flow out of the uterine cavity and ensuring sustained action; (2) Biological scaffold: under scanning electron microscopy, PRF exhibits a three-dimensional network structure with fibrin serving as the skeletal framework. This unique architecture promotes endometrial migration and regeneration, making it an ideal biologically active scaffold for facilitating the migration of new cells [15,16]. Studies have reported that PRF demonstrates favorable adhesive properties for bone marrow mesenchymal cells, which, in turn, promote cell proliferation, tissue formation, and expedited wound healing [17]. The current form of PRF available is in the form of a gel, which poses a limitation as it cannot be directly injected. This poses a challenge when using PRF as a standalone treatment or in combination with other biomaterials for various medical applications. However, researchers are actively exploring innovative techniques to overcome this limitation and enhance the clinical utility of PRF in diverse medical contexts [18].

Recent studies have produced new techniques for the liquid PRF variant known as PRF lysate, which is also a blood gel derivative. PRF lysate is a component of a PRF that also includes glycan chains, structural glycoproteins, and cytokines, all of which act as natural angiogenesis initiators. In the previous study, Anitua et al. (2010) claimed that the lysate form has a higher concentration of growth factors than PRF gel [19]. This liquid form of PRF is produced by activating platelets using temperature and sonication [6,7,20]. Lele Ma et al. (2021) conducted a study that demonstrated the positive effects of PRF exudates collected from a culture medium on the proliferation and migration of endometrial stromal cells (ESC) in vitro. Additionally, Lele Ma et al. found that the transplantation of PRF gel had beneficial effects on the structure of the uterus and the regeneration of the endometrial luminal epithelium and endometrial glands in vivo [21]. The application of PRF gel demonstrated a therapeutic effect on thin endometrium in rats, increasing endometrium thickness and enhancing marker expression, indicating potential for treating a thin endometrium [22]. Moreover, the efficacy and safety of autologous PRF gel for the treatment of infertility with intrauterine adhesions were identified [23]. The use of PRF lysate has not been widely publicized in vitro and in vivo, despite the fact that PRF is already employed in the wound healing field [6]. 

In vitro model systems provide several advantages, including the ability to manipulate experimental variables, reduction of ethical concerns associated with animal experimentation, and the potential for high-throughput screening of interventions. The application of in vitro model systems offers researchers valuable tools for studying the intricate interactions between embryos and the endometrium. These model systems afford the opportunity to explore the intricate molecular mechanisms that dictate embryo implantation and pregnancy. The continuous advancements in these model systems hold promise for the development of innovative diagnostic and therapeutic approaches for infertility, ultimately leading to improved outcomes in assisted reproductive technologies [24].

This study aimed to evaluate the wound healing and regenerative potential of PRF lysate collected by incubating PRF gel at 37 °C for 24 h and PRF gel in mice in vitro through the release of growth factors, endometrial structure, and function by embryo implantation in vitro. The findings of this study are experimental evidence that can be applied to clinical endometrial repair procedures.

## 2. Results and Discussion

### 2.1. Growth Factors Quantification

**Figure 1:**
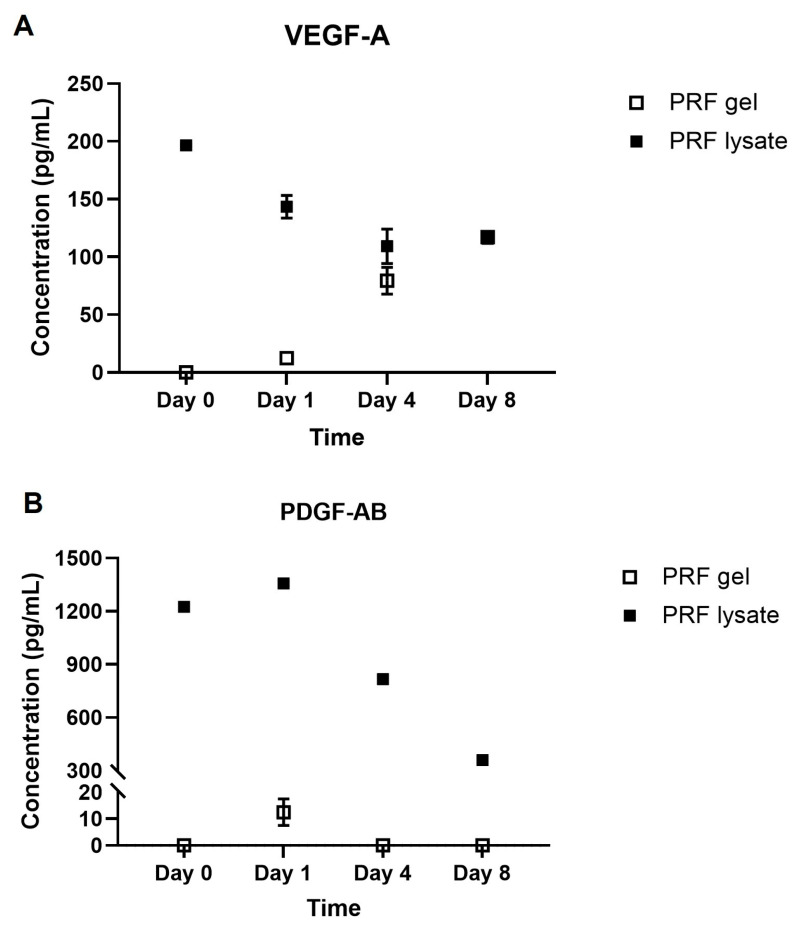
Growth factor concentrations over time: (**A**) VEGF-A concentration; (**B**) PDGF-AB concentration.

This study focused on the determination of two released growth factors, namely VEGF-A and PDGF-AB, from PRF gel and PRF lysate. The growth factors were quantified for their concentrations at four time points (0 h, 24 h, 4 days, and 8 days). The results showed that only the highest PRF lysate group’s VEGF-A concentration was observed at the beginning, at 196.50 ± 0.97 pg/mL (*p* < 0.05). In this group, the concentration of these growth factors was prone to decrease with time. At days 4 and 8, respectively, the lowest values were 109.20 ± 14.93 pg/mL and 115.80 ± 2.96 pg/mL (*p* > 0.05). In contrast to PRF lysate, the concentration of this growth factor increased over time in the PRF gel group (*p* < 0.05). The maximum level of VEGF-A in this group at the end of the survey was 117.20 ± 0.62 pg/mL, which was identical to the level at days 4 and 8 in the PRF lysate group (Figure 1A).

Regarding PDGF-AB, at the starting time, the concentration of this growth factor in PRF lysate was 1224.67 ± 16.18 pg/mL, followed by a statistically significant increase to 1358.00 ± 12.99 pg/mL after 24 h (*p* < 0.05). However, PDGF-AB concentration rapidly decreased to 816.30 ± 15.31 pg/mL at day 4, and this value continued to decrease at day 8, which was 360.80 ± 15.63 pg/mL (*p* < 0.05). Only the PDGF-AB concentration at 24 h after incubation was 12.50 ± 5.00 pg/mL in the PRF gel, as opposed to the PRF lysate. (Figure 1B).

Platelet-derived growth factor (PDGF) and vascular endothelial growth factor (VEGF) are growth factors found in PRF. Numerous studies have discovered endometrial VEGF expression throughout the menstrual cycle, including a notable increase in the secretory phase, pointing to a potential role for endometrial VEGF during embryo implantation. In conjunction with the elevation of VEGF, the glandular epithelium’s VEGFR-1 and VEGFR-2 are also raised during the secretory phase [25,26,27]. The secretory phase is characterized by the presence of epithelium with simple pseudostratified columnar-shaped cells. Previous studies have highlighted the diverse roles of PDGF in cellular growth, angiogenesis, inflammation, and tissue healing. In the context of the endometrium, PDGF has been shown to induce cell proliferation in various cell types, including endometrial stromal cells, decidual cells, and endometrial epithelial cells. These findings suggest that PDGF plays a crucial role in the processes occurring in the endometrium during the secretory phase, potentially contributing to the preparation of the uterine lining for embryo implantation [28].

ELISA results regarding VEGF-A and PDGF-AB showed that the treatment biomaterials, including PRF gel and PRF lysate, could release growth factors over time during the incubation. However, the concentrations of these growth factors in the PRF lysate were found to be higher compared to the PRF gel. Furthermore, it was observed that the concentration of PDGF-AB decreased more rapidly compared to VEGF-A. By being squeezed from PRF gel, PRF lysate could contain circulation plasma adhesive proteins, such as fibrinogen, fibronectin, vitronectin, and thrombospondin [29]. Later, these activated proteins play a crucial role in the activation of platelets, leading to the formation of fibrin clots. These fibrin clots act as a matrix for the release of additional growth factors, further promoting the process of wound healing in the early stages. The combined action of these proteins and growth factors creates a favorable environment for tissue regeneration and repair, aiding in the healing process [30]. Indeed, the preservation of growth factor concentration in PRF lysate over time can be attributed to the activation of platelets and the formation of fibrin clots. However, it is important to note that the fibrin structure in the PRF lysate group was found to be significantly looser compared to the PRF gel group. This structural difference may contribute to the rapid decrease in growth factor levels over time in the PRF lysate group. Furthermore, the sustained levels of released VEGF-A and PDGF-AB may be influenced by their respective binding affinities to fibrin and fibrinogen, as well as their half-life times ex vivo. These factors can affect the release trends of the growth factors throughout the eight days of incubation. In the case of VEGF, previous studies have shown that it exhibits a high binding affinity to fibrin and fibrinogen, as well as a long half-life time. These characteristics may contribute to the sustained levels of VEGF-A in the PRF lysate group during the incubation period [31,32]. 

### 2.2. Histological Staining

**Figure 2:**
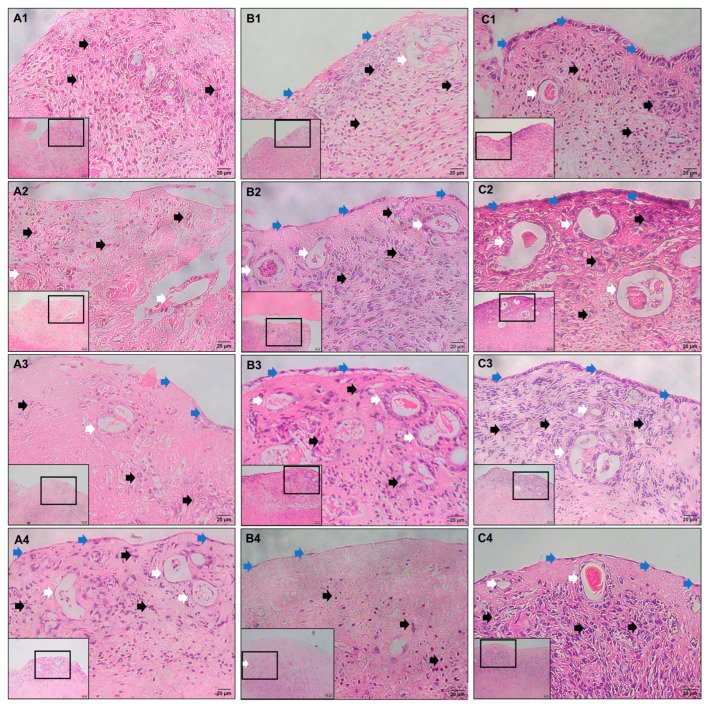
Uterine structure in the groups after treatment during cultural times (×200, ×400): (**A1**–**A4**) non-treated tissues at Day 4, Day 8, Day 12, and Day 16, respectively; (**B1**–**B4**) PRF gel-treated tissues at Day 4, Day 8, Day 12, and Day 16, respectively; (**C1**–**C4**) PRF lysate-treated tissues at Day 4, Day 8, Day 12, and Day 16, respectively. Blue arrow: luminal epithelial cells; white arrow: uterine glands; black arrow: stromal cells.

Endometrial histological evaluation was performed in three groups, including the control group, the PRF gel group, and the PRF lysate group, over a period of 16 days of treatment. The findings indicated that endometrial wound healing and regeneration occurred in all three groups. However, the timing of regenerative initiation, impact, and maintenance varies among groups (Table 1, Figure 2). Specifically, the PRF lysate group exhibited early initiation of regenerative processes, with the presence of uterine glands and luminal epithelial cells observed as early as 4 days following treatment. In contrast, this regenerative phenomenon occurred later in the control and PRF gel groups, with regeneration observed at days 8 and 12, respectively. Furthermore, the results revealed that the tissue structure was most recovered at day 8 in the PRF lysate group. However, it is important to note that degradation of endometrial tissues was observed in all three groups, indicating the limitations of in vitro culture conditions. These findings suggest that PRF lysate has a more pronounced effect on stimulating endometrial wound healing and regeneration compared to PRF gel and the control group.

Histological results demonstrated that, on day 8 of culture, the uterine structure was full of endometrial and epithelial cells, showing a pseudostratified morphology consistent with the implantation function of the embryo. Therefore, the time point of day 8 of post-treatment culture was selected to evaluate pinopode expression and embryo implantation function.

The findings of this study revealed that injured mouse endometrial tissues exhibited the ability to undergo self-renewal in vitro, as evidenced by the development of a surface epithelium and glandular system. However, the efficiency of tissue restoration was significantly enhanced when stimulated with PRF lysate. In contrast, the impact of PRF gel on tissue restoration was less pronounced. Furthermore, the form of PRF gel may serve as a beneficial barrier, effectively separating the healing area from the external environment, which may not promote optimal healing. On the other hand, PRF lysate, which is a component of PRF, contains various bioactive components, such as glycanic chains, structural glycoproteins, and cytokines. These natural angiogenesis initiators likely contribute to the enhanced restoration of injured tissues [7]. The action of PRF gel and PRF lysate resulted in significant changes in the endometrium. In both groups, the injured endometrial epithelium was restored following treatment. However, it was observed that only in the PRF lysate group, there were notable morphological changes in the endometrial cells. The luminal epithelial cells exhibited a reduction in microvilli and displayed a pseudostratified structure. Additionally, the secretion of the uterine glands was also observed. These changes in the endometrium resemble the characteristics typically seen during the early secretory phase, when the functional endometrium is prepared for embryo implantation. This suggests that the application of PRF lysate may induce an accelerated regenerative response in the endometrium, promoting the development of a receptive environment for successful embryo implantation [33]. Overall, these results highlight the potential therapeutic benefits of PRF lysate in promoting tissue regeneration and angiogenesis in injured endometrial tissues.

### 2.3. Scanning Electron Microscope

**Figure 3:**
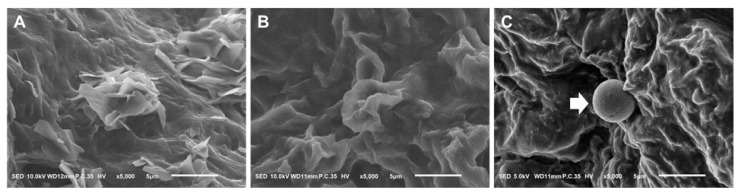
The presence of pinopodes in the groups (×5000): (**A**) Non-treated tissues at Day 8; (**B**) PRF gel-treated tissues at Day 8; (**C**) PRF lysate-treated tissues at Day 8. Pinopode: white arrow.

In both rodents and humans, pinopodes emerge from the luminal epithelium of the uterus’ apical surface during the window of receptivity. The best way to see this is through a scanning electron microscope (SEM). The results showed that the surface of uterine tissues treated with PRF lysate exhibited smooth mushroom- or balloon-shaped projections that emerged from the apical surface of the endometrium. This phenomenon was not observed in the control (non-treated) and PRF gel groups (Figure 3).

Precise synchronization between the fetal-derived trophoblast cells and the maternal uterine luminal epithelium is necessary for successful embryo implantation. It is established that the pinopode’s presence supports process control. This study demonstrated that pinopode, which was a particular receptor of the “implantation window” with a smooth mushroom- or balloon-like shape and full protrusion, was expressed in the endometrium treated with PRF lysate [34].

According to their ultrastructural morphology, pinopodes are commonly categorized as developing/immature, completely developed/mature, and regressing based on a scanning electron microscopy (SEM) study of pinopodes during the estrus cycle. The size of pinopodes ranges from 1.0 to 10.0 µm, with prior average values being about 4.0 and 6.0 µm in rats and mice, respectively [35]. In this study, the size of pinopodes observed in the PRF lysate group was smaller than the size of mature pinopodes. Although the pinopodes in the PRF lysate group displayed a characteristic mature morphology, their size was not appropriate. Therefore, it is important to evaluate several molecular markers associated with the presence of pinopodes, including integrins, LIF, L-selectin, HOXA10, Glrx, GlyA, HB-EGF, osteopontin, and mucins, to provide more convincing evidence and further understand the impact of PRF lysate treatment on pinopode development and functionality [36].

### 2.4. Embryo Implantation

**Figure 4:**
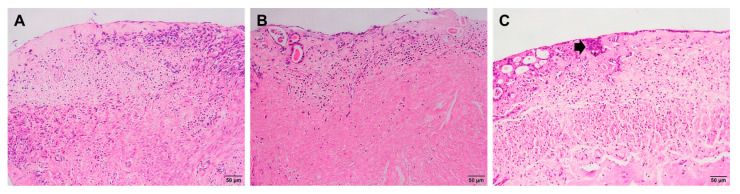
Embryo implantation at day 4 after transfer (×200): (**A**) Non-treated tissues; (**B**) PRF gel-treated tissues; (**C**) PRF lysate-treated tissues. Black arrow: embryo.

After 4 days from the time of embryo transfer, histological structures were observed to determine the location of embryo implantation on the endometrium. There were no embryos recognized on the surface of injured endometrium without treatment (Figure 4A), as well as in the PRF gel group (Figure 4B). Meanwhile, in the PRF lysate group, the embryo reached the invasion phase, with the embryo entirely lying in the stroma of the functional zone (Figure 4C).

Recent studies on mice have demonstrated that the processes of embryo implantation involve three steps: invasion, attachment, and uterine receptivity. Embryo invasion requires the availability of uterine stroma and luminal epithelium [37]. Consequently, the outcomes of the embryo’s invasion into the endometrium in the PRF-lysate group confirmed the function of stimulation in facilitating the endometrial embryo’s implantation.

As a result, this study demonstrated that PRF lysate, which was made by activating PRF gel for 24 h at 37 °C, could induce endometrial regeneration more effectively than PRF gel. However, different lysate preparation methods have not been performed, and their effects have not been compared. Therefore, the study could not confirm that this PRF lysate is the most effective in promoting endometrial wound healing and regeneration.

To date, there have been no studies assessing the efficacy of treatments on in vitro models of damaged uterine tissue. This is primarily due to the lack of a suitable long-term tissue culture model that has been established. However, PRF gel, one of the blood gel derivatives, was evaluated for its effect on endometrial function in animal models and clinical trials. Intrauterine administration of PRF was demonstrated to stimulate damaged endometrium regeneration in rats [21]. Moreover, PRF was placed into the uterine cavity of infertility patients with intrauterine adhesions (IUAs), leading to an increase in the pregnancy rate [23].

## 3. Conclusions

PRF gel and PRF lysate derived from mouse blood release VEGF-A and PDGF-AB over time. PRF lysate has higher concentrations of growth factors and greater regenerative effectiveness compared to PRF gel. While both promote endometrial structure restoration, only PRF lysate can fully restore injured endometrium morphology, slow fibrosis progression, and support pinopode expression as well as embryo implantation.

## 4. Materials and Methods

### 4.1. Animals

Physiologically healthy female mice (Mus musculus var. Albino) were 8–12 weeks old. All mice were maintained under controlled temperature and light conditions and frequently provided free food and water at the Laboratory of Tissue Engineering and Biomedical Materials, University of Science, Vietnam National University, Ho Chi Minh City, Vietnam. The research was approved by the Animal Care and Use Committee, University of Science, VNU-HCM (580B/KHTN-ACUCUS). A total of 42 mice were used in this study, including 15 mice for blood collection and 27 mice for tissue collection.

### 4.2. PRF Gel and PRF Lysate Preparation

Mice were anesthetized, and blood was drawn from their hearts using a 26-gauge needle. After that, blood was transferred into a 15-mL centrifuge tube (Nunc, Waltham, Massachusetts, USA) and centrifuged for 15 min at 2500 rpm at room temperature without anticoagulants (Hettich, Föhrenstraße 12, 78532 Tuttlingen, Germany). The PRF gels were then removed from the red blood cell layer by using sterile scissors and forceps and transferred into 1.5-mL centrifuge tube (Eppendorf, Germany). For PRF lysate preparation, the PRF gels were put in the incubator (Panasonic, Japan) at 37 °C for 24 h, and the PRF lysate was collected by using sterile forceps to squeeze the gels. PRF lysate was preserved at −20 °C for the following usages. The PRF gel was collected at the time of use.

### 4.3. Growth Factor Quantification

To evaluate the release of growth factors, PRF lysates were used directly, and extracts of PRF gels were collected according to ISO 10993-12 [38]. There are four time points that were determined, including the starting time (0 h), day 1 (D1), day 4 (D4), and day 8 (D8) after incubating at 37 °C, 5% CO_2_. An aliquot of the PRF lysates and the extract of the PRF gel were collected for enzyme-linked immunosorbent assay (ELISA) testing to determine the amount of VEGF-A (RAB0509, Sigma-Aldrich, Burlington, MA, USA; sensitivity: 2 pg/mL, standard curve range: 4.1–1000 pg/mL) and PDGF-AB (RAB1857, Sigma-Aldrich, Burlington, MA, USA) concentration according to the manufacturer’s instructions. An amount of 100 µL of the samples was placed in the corresponding wells of the 96-well plate. The plate was covered and incubated overnight at 4 °C with gentle shaking. Following the removal of the solution from this plate, each well was thoroughly washed four times with 300 µL of 1× wash solution. After that, 100 µL of detection antibody solution was added to each well and was left to sit for an hour at room temperature. Then, wells were rinsed four more times, given a 100 µL addition of streptavidin solution, and allowed to incubate for 45 min. Following a further four times of washing, 100 µL of TMB solution was added to each well and was incubated for 30 min in the dark. The final addition was 50 µL of stop solution. Absorbance was assessed using a microplate reader at 450 nm (Biochrom EZ Read 400, Holliston, MA, USA). This experiment was repeated at least 3 times.

### 4.4. Establishment of Mouse Injured Tissue Models

The diestrus phase mice, which were identified through vaginal smear cytology, were simultaneously triggered to reach estrus with 5 IU PMSG (ProSpec, Saint Louis, MI, USA) and 5 IU hCG (LG Life Science, Seoul, Republic of Korea) sequentially. The uterus was collected after 16 h following the secondary hormone dose. After anesthesia and cervical dislocation, an incision was made vertically and horizontally in the mouse’s lower abdomen to expose internal organs. All internal organs were put aside to locate both uterine horns. The uterine horns were then separated from the ovaries, and adipose and connective tissues were extracted using sterile scissors and forceps. Following this, uterine horns were separated half along their length from the uterine fundus before being transferred into a 35-mm dish (Nunc, Rochester, NY, USA) containing 3 mL of sterile washing solution (Global Collect, LifeGlobal, Guelph, Canada). These collected uterine horns were thoroughly washed with washing solution in the class II biosafety cabinets (ESCO, Singapore) to remove all blood and fat. Next, they were transferred into a petri dish and moderately sprayed with a washing solution to prevent surface dehydration. Under a stereo microscope (Zeiss SteREO Discovery.V8, Oberkochen, Germany), the process of uterine damage induction was controlled. Forceps were used to stabilize a uterine horn, and a thin, sharp pair of bending scissors was used to gradually remove the functional zone of the uterus. Following the process, all injured horns were cut into small endometrial tissues measuring 2× 2 mm.

### 4.5. PRF Gel and PRF Lysate Treatment on Mouse Injured Tissue Models

Briefly, mouse injured endometrial tissues were divided into three groups: a control group (injured endometrium tissues without treatment) and two experimental groups (injured endometrium tissues with treatments including PRF gel and PRF lysate, separately). The tissues were placed in the wells of cell culture insert plates (Nunc, Rochester, NY, USA), with the endometrium facing up. Each well contained 1 mL of culture medium (DMEM-F12 (Sigma-Aldrich, Burlington, MA, USA), containing 20% fetal bovine serum (Sigma-Aldrich, Burlington, MA, USA), 10 nM estradiol (Sigma-Aldrich, Burlington, MA, USA), 100 nM progesterone (Sigma-Aldrich, Burlington, MA, USA), 10 ng/mL EGF (Sigma-Aldrich, Burlington, MA, USA), 1% ITS (Sigma-Aldrich, Burlington, MA, USA), and 1% penicillin/streptomycin (Sigma-Aldrich, Burlington, MA, USA). PRF gel and PRF lysate were added to cover the endometrial apical surface with a volume of 5 µL for each uterine tissue. The culture plates were then placed in the incubator at 37 °C in a fully humidified atmosphere of 5% CO_2_. The medium was changed every two days, with 500 µL of the old culture medium being replaced. The culture process for each group lasted for 16 days and was assessed at four different time points: 4 days of culture (D4), 8 days of culture (D8), 12 days of culture (D12), and 16 days of culture (D16).

### 4.6. Histological Staining

The biopsy samples were embedded in paraffin, cut into 5 µm thick slices, and stained with hematoxylin–eosin (H&E) (Sigma-Aldrich, Burlington, MA, USA) after being fixed for 24 h in 10% formalin. By utilizing H&E staining, endometrial morphology was examined, and pictures were taken with an inverted microscope (Olympus Co., Tokyo, Japan). This experiment was assessed at all four time points.

### 4.7. Scanning Electron Microscope

Cultured endometrial tissues from the PRF gel-treated group, the PRF lysate-treated group, and the non-treated group, which were harvested at the greatest point of tissue restoration based on histological assessment, were washed with deionized water, and fixed in SEM solution fixative for six hours. These tissues completely sank in the SEM fixative. After six hours, tissues were thoroughly cleaned with deionized water (Sigma-Aldrich, Burlington, MA, USA) and left to be completely dried. Following this, tissues were frozen at −86 °C and freeze-dried before being sprayed with gold for a scanning electron microscope (JEOL, Tokyo, Japan). The morphology of the tissue surface was observed to demonstrate pinopode expression.

### 4.8. Embryo Implantation

Female mice were induced to hyperovulate with 10 IU of PMSG and hCG injections and mated in a 2:1 ratio with male mice. Embryos were collected on day 4.5 after ovulation, which coincides with the greatest point of tissue restoration based on histological assessment. Following the operation and collection of the mouse uterine horn, a 1-mL needle filled with embryo collection solution was gently inserted into a uterine branch to flush out any embryos present. Embryos were collected in a 35-mm dish and detected under an inverted microscope. Embryos were applied to the surface of tissues from both the treated and non-treated groups. These tissues were cultured for another four days before being fixed in 10% formaldehyde (Merck, Darmstadt, Germany) at 4 °C for a duration of 12 h for H&E staining.

### 4.9. Statistical Analysis

Statistical analysis of ELISA results for VEGF-A and PDGF-AB releases was con-ducted using SPSS software. The results were presented as the mean ± standard deviation (mean ± SD). Group comparisons were performed using one-way ANOVA, where the independent variable was the growth factor, and the goal was to investigate if different levels of the growth factor had a measurable effect on the dependent variable (time). A significance level of *p* < 0.05 was considered statistically significant.

## Figures and Tables

**Table 1:** Endometrial histological results in all groups after treatment.

Time	Groups		
	Non-Treated (Control)	PRF Gel-Treated	PRF Lysate-Treated
Day 4	− Luminal epithelial cells and uterine glands were not observed (Figure 2(A1)) − The stromal cells were irregularly dispersed and had an oval shape (Figure 2(A1), black arrow)	− Several luminal epithelial cells were observed (Figure 2(B1), blue arrow) − Appearance of several gland-like structures with elongated glandular epithelial cells (Figure 2(B1), white arrow) − Elongated stromal cells were densely distributed in the upper layer of the endometrium (Figure 2(B1), black arrow)	− A single layer of columnar epithelial cells with apical microvilli (Figure 2(C1), blue arrow) − The appearance of endometrial glands with elongated and columnar epithelial cells and the secretion of uterine glands were observed (Figure 2(C1), white arrow) − Elongated stromal cells were densely distributed in the upper layer of the endometrium (Figure 2(C1), black arrow)
Day 8	− Luminal epithelial cells were not observed (Figure 2(A2)) − Appearance of several gland-like structures with elongated epithelial cells (Figure 2(A2), white arrow) − The stromal cell shape and distribution resembled those of day 4 (Figure 2(A2), black arrow)	− Elongated luminal epithelial cells had a wider coverage (Figure 2(B2), blue arrow) − Several columnar glandular epithelial cells were observed (Figure 2(B2), white arrow) − The stromal cell shapes were oval and elongated (Figure 2(B2), black arrow).	− Pseudostratified columnar epithelium was identified (Figure 2(C2), blue arrow) − Columnar epithelium and secretion of the uterine gland were observed (Figure 2(C2), white arrow) − The stromal cells were predominantly oval in shape and uniformly distributed (Figure 2(C2), black arrow)
Day 12	− Several elongated luminal epithelial cells were observed (Figure 2(A3), blue arrow) − The state of gland and stromal cells resembled those of day 8 (Figure 2(A3), white and black arrow)	− Luminal epithelial cells displayed an elongated shape (Figure 2(B3), blue arrow) − Columnar epithelium and secretion of the uterine gland were observed (Figure 2(B3), white arrow) − The stromal cell shapes were oval and elongated (Figure 2(B3), black arrow)	− Apical microvilli were observed on the surface of the luminal epithelium, similar to day 4 (Figure 2(C3), blue arrow) − The state of gland and stromal cells resembled those of day 8 (Figure 2(C3), white and black arrow)
Day 16	− Degradation of endometrial tissues (Figure 2(A4,B4,C4))		

## Data Availability

Publicly available datasets were analyzed in this study. This data can be found here: https://www.dropbox.com/scl/fi/qwiz61qimn1l2jql8qmfl/ELISA-Test-of-GFs-In-PRF-groups.xlsx?rlkey=c4ylslzqozbxw7zsi3g46q5gz&dl=0 (accessed on 27 August 2023).
